# Probing electronic structure in berkelium and californium via an electron microscopy nanosampling approach

**DOI:** 10.1038/s41467-021-21189-1

**Published:** 2021-02-11

**Authors:** Alexander Müller, Gauthier J.-P. Deblonde, Peter Ercius, Steven E. Zeltmann, Rebecca J. Abergel, Andrew M. Minor

**Affiliations:** 1 National Center for Electron Microscopy, Molecular Foundry, Lawrence Berkeley National Laboratory, Berkeley, CA USA; 2 Department of Materials Science and Engineering, University of California, Berkeley, CA USA; 3 Chemical Sciences Division, Lawrence Berkeley National Laboratory, Berkeley, CA USA; 4 Glenn T. Seaborg Institute, Physical & Life Sciences, Lawrence Livermore National Laboratory, Livermore, CA USA; 5 Department of Nuclear Engineering, University of California, Berkeley, CA USA

**Keywords:** Solid-state chemistry, Nanoparticles, Characterization and analytical techniques, Imaging techniques, Nanoparticles

## Abstract

Due to their rarity and radioactive nature, comparatively little is known about the actinides, particularly those with atomic numbers higher than that of plutonium, and their compounds. In this work, we describe how transmission electron microscopy can provide comprehensive, safe, and cost-effective characterization using only single nanogram amounts of highly-radioactive, solid compounds. Chlorides of the rare elements berkelium and californium are dropcast and then converted in situ to oxides using the electron beam. The f-band occupancies are probed using electron energy loss spectroscopy and an unexpectedly weak spin-orbit-coupling is identified for berkelium. In contrast, californium follows a *jj* coupling scheme. These results have important implications for the chemistries of these elements and solidify the status of californium as a transitional element in the actinide series.

## Abstract (web summary)

The obtention and study of actinide elements is challenging due to various factors including their radioactivity and scarcity. Herein, the authors characterize the atomic and electronic structure of Am, Cm, Bk, and Cf compounds using a transmission electron microscopy-based workflow that only requires nanogram amounts of the actinide element.

## Introduction

Many radioactive materials have unique properties and several of them play central roles in applications such as nuclear energy and cancer therapy^1–5^. However, the health hazards posed by radioactive elements have always been a major hurdle to their research. As a result, comparatively little is known about them and their chemistries. Within the actinide series from actinium (Ac) to lawrencium (Lr), of which all isotopes are radioactive, the vast majority of research has been on uranium (U), thorium (Th), neptunium (Np), and plutonium (Pu). Further, of all described actinide-containing crystal structures (~7000), only about 0.5% include elements with atomic numbers greater than that of Pu^6^. This imbalance can in part be explained by the relevance of the early actinides to nuclear power, space exploration, and nuclear weapons programs^1–5^, in part by the natural occurrence of thorium and uranium, on which much research has been focused. Another key factor is the extreme challenges introduced when working with the heavy actinides. Transplutonium elements are without exception synthetic elements and their production requires long irradiation campaigns in high neutron flux nuclear reactions, followed by arduous purification steps performed in hot cells and using remote handling techniques^7^. These processes are time-consuming, difficult, and therefore inherently expensive, yet yield only minute amounts of the targeted isotopes. The current global production capabilities are milligram amounts of berkelium-249 (^249^Bk) and californium-249 (^249^Cf), sub-microgram amounts of einsteinium-254 (^254^Es), and picogram-scale quantities of fermium-257 (^257^Fm)^7^. Furthermore, all transcurium elements except for Cf (^249^Cf, half-life ≈ 351 years) that are currently available in appreciable quantities only have relatively short-lived isotopes, which rapidly decay and emit large amounts of radiation. This prevents stockpiling and increases the logistical burden even further. Considerable efforts have therefore gone into minimizing the amounts required for experiments^8,9^, but typical characterization techniques, such as X-ray diffraction and X-ray absorption spectroscopy still require microgram to milligram amounts per sample^10,11^. In the case of transplutonium elements, even such small amounts can be cost-prohibitive, pose significant health hazards and necessitate large administrative and experimental controls. Only a few institutions worldwide are set up to tackle these challenging requirements, and even then, experiments can still fail due to self-irradiation-induced artifacts^12–14^.

In light of these challenges, the use of transmission electron microscopy (TEM), a technique allowing detection and investigation of even single atoms, seems like a promising approach^15–20^. Yet, we are only aware of two research groups that have used TEM to study transplutonium elements. In the 1970’s, a group at the Oak Ridge National Laboratory used TEM to solve crystal structures from polycrystalline diffraction patterns^13,14,21^ and in the 2000’s, a group at Lawrence Livermore National Laboratory used electron energy loss spectroscopy (EELS) to investigate electronic structures^22–27^. Neither group took advantage of the method’s small detection limit but either worked with the maximum amounts available to them or started from a bulk sample, which was then prepared for TEM using traditional sample preparation methods^23^. Recent improvements have made TEM significantly more powerful and it can now even be used to investigate single atoms.

Here, we leverage these improved capabilities and complement them with a workflow that allows working with amounts as small as a single nanogram to explore crystal and electronic structures of Bk and Cf oxides. Both elements are exceedingly rare and expensive and their high radioactivity poses a serious health risk. Further, ^249^Bk (half-life ≈ 330 days) β^−^-decays to ^249^Cf. Samples thereby self-contaminate and experiments benefit from a fast workflow and, when possible, processing of the samples close to the characterization facility. TEM provides a way to analyze morphology, crystal structure, and electronic structure in a safe, cost-effective, and rapid manner. EELS allows investigating the f-band occupancies of Bk- and Cf-compounds and an unexpectedly weak spin–orbit coupling is determined for Bk.

## Results

### Sample preparation

**Fig. 1:**
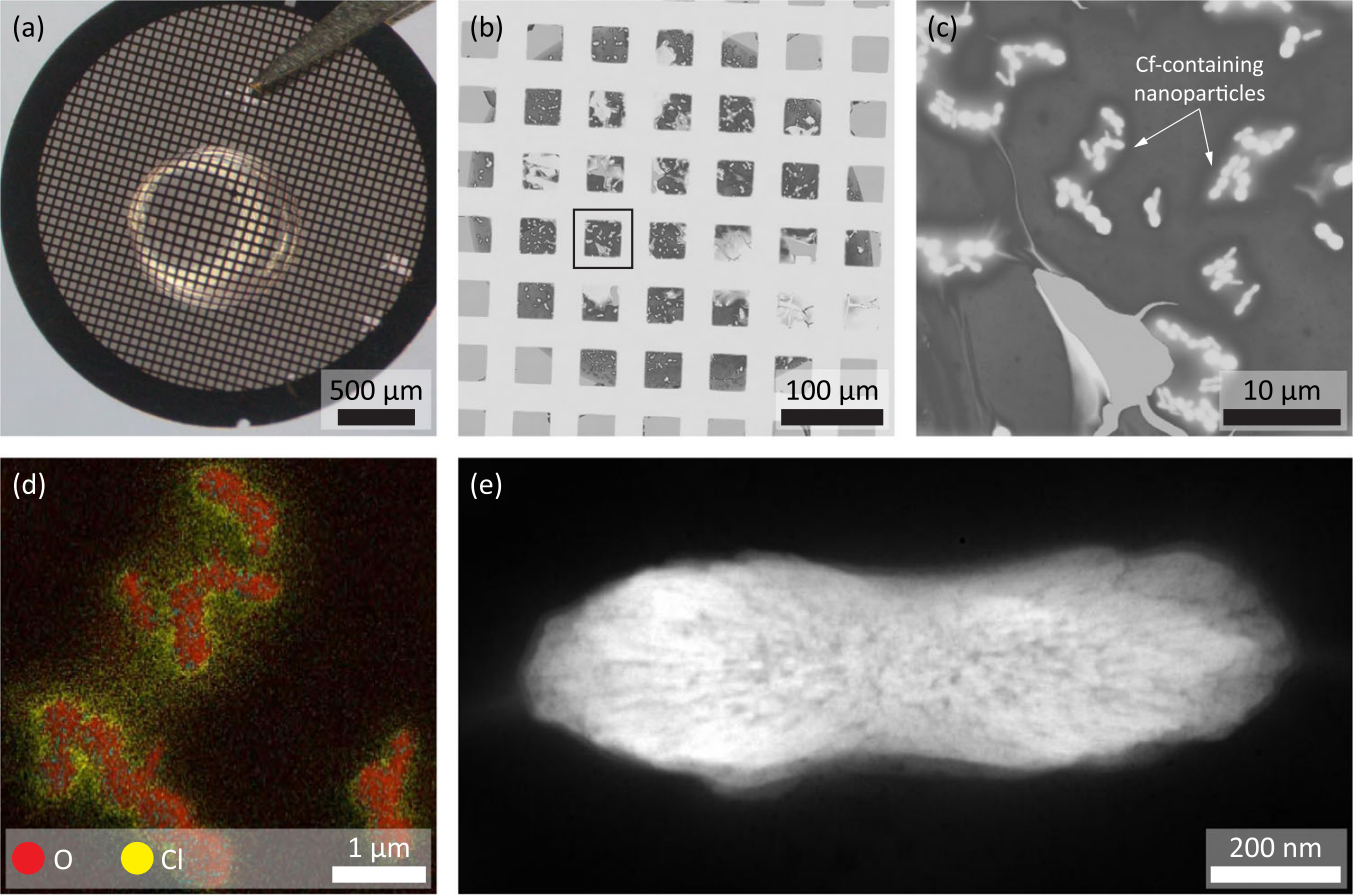
Investigation of a dropcast Cf-containing sample. **a** Droplet of a solution containing Cf on a TEM grid. **b** The dried drop is imaged using the STEM detector of an SEM. A box marks the region shown in (**c**), in which individual particles can be seen. **d** EDS mapping, with O marked in red and Cl marked in yellow, confirms that the particles contain O and have Cl-enriched surfaces. Individual spectra also confirm that the particles contain Cf (Supplementary Fig. 5). **e** HAADF-STEM image of individual particles shows the pores.

TEM samples of actinide compounds were prepared by dropcasting aqueous acidic solutions of the chloride salts onto the carbon-film side of a standard TEM grid and letting the samples dry at room temperature and ambient pressure (Fig. 1 and Supplementary Fig. 1). Each drop had a volume smaller than 1 μl and contained between 1 and 10 ng of the actinide. As the liquid evaporated and the drop contracted, the concentration of the solute in the drop rose until it supersaturated and particles precipitated. In an idealized case, one would expect even contraction of the drop and, ultimately, material deposition in a tiny area of the TEM grid. In practice, contact-line-pinning was a dominant factor that caused uneven contraction of the drop and the precipitation of particles on geometrical features (Supplementary Fig. 1). Prior to working with rare, radioactive elements, we validated the preparation approach using drops containing 1 ng SmCl_3_, a non-radioactive surrogate with respect to ion size^28^ and solubility^29^. This yielded a carpet of evenly distributed, presumably hydrated, SmCl_3_ particles with well-defined facets (Supplementary Fig. 2).

We then moved on to experiments with the isotopes ^243^Am, ^248^Cm, ^249^Bk, and ^249^Cf. In contrast to the preliminary experiments, none of the actinides deposited as neatly. We always found large, mostly electron-opaque deposits in the middle of the deposition area, which were identified as non-radioactive contaminants (Supplementary Figs. 3 and 4) using energy-dispersive X-ray spectroscopy (EDS) and EELS. The presence of these non-radioactive contaminants in actinide samples reveals another challenge unique to working with actinides: Their production is difficult and often focused on radiopurity rather than chemical purity. The detection of these previously overlooked, non-radioactive contaminants highlights the critical information that can be harvested via electron microscopy and could have large implications on the quality control of rare isotope production and nuclear forensics.

### Structures of Cf- and Bk-containing particles

**Fig. 2:**
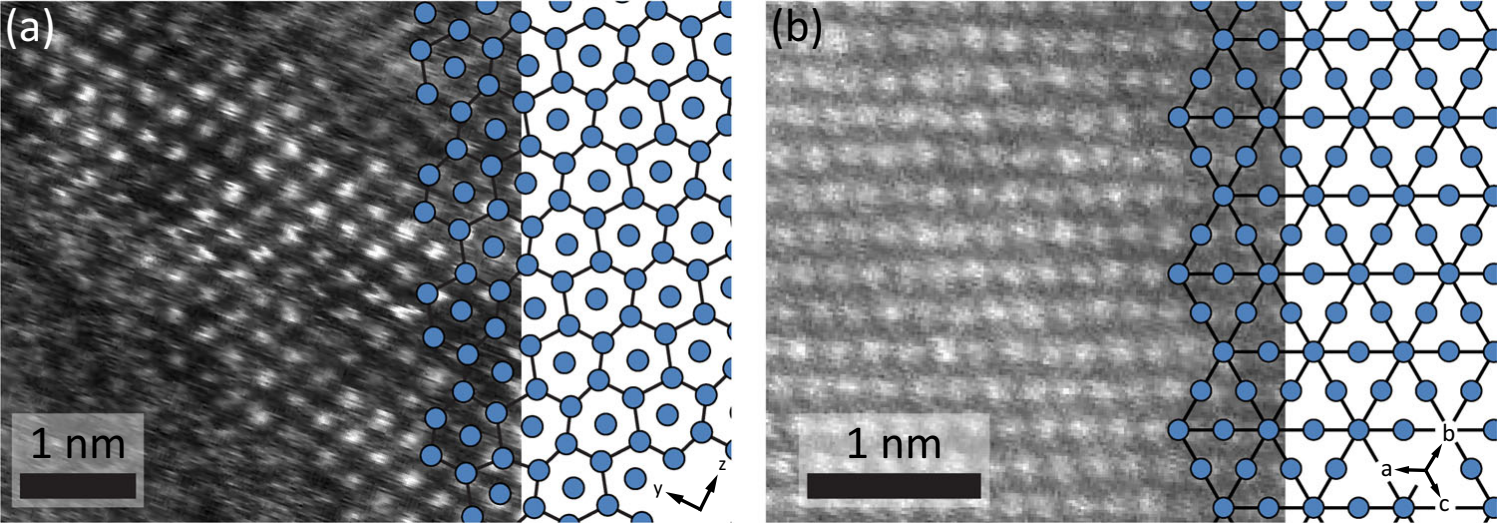
Crystal structures of Cf_2_O_3_ and BkO_2_. **a** Cf_2_O_3_ viewed along the [100] lattice vector and partly overlaid schematic showing Cf columns. **b** BkO_2_ viewed along the [111] lattice vector with Bk atoms shown in blue in the partly overlaid schematic. Please note that O atoms are not visible in the HAADF-STEM images and are therefore not shown in the schematic, either. In the schematics, atoms were connected by lines to accentuate structural motifs.

Cf-containing particles precipitated in a large area and in large amounts. These particles formed with a distinct, dumbbell-like morphology several micrometers in length and had a structure with pores along the long axis of the particle (Fig. 1e). Spectroscopic analysis confirmed the presence of Cf throughout the particle. It also indicated the presence of oxygen (O) throughout the particle and of chlorine (Cl) at the surfaces (Fig. 1d). The particles were beam-sensitive and it was not possible to accurately determine their chemical composition or crystal structure. However, strong Cl, O, and Cf signals in EDS spectra suggested the formation of a hydrated chloride material (Supplementary Fig. 5a), which is in line with the use of a solution of californium chloride in hydrochloric acid. After extended electron-beam irradiation, a beam-stable, polycrystalline compound formed in situ which had strong Cf and O signals in EDS and EELS, but no signs of Cl (Supplementary Fig. 5b). Electron diffraction proved challenging due to crystallite size and nearby contaminants, but the structure of the compound as seen in atomically-resolved high-angle annular dark-field (HAADF) STEM images was consistent with trigonal Cf_2_O_3_ (Fig. 2a)^30^. Cf_2_O_3_ has in the past been prepared by heating CfOCl in water vapor or O_2_^31^, and we propose that the electron-beam irradiation^32^ had a similar effect.

In comparison, the sample prepared using the Bk(III) stock solution was significantly more contaminated to the point that contaminants, particularly carbon (C), dominated. However, we were able to directly observe nanoparticles of cubic BkO_2_ (Fig. 2b) instead of needing extensive electron-beam irradiation^33^. We assume that the electron-beam-induced reaction happened too quickly to witness, as the direct formation of BkO_2_ from an aqueous solution is thermodynamically unfavorable. Nonetheless, the beam-induced formation of BkO_2_, with tetravalent Bk, as opposed to Bk_2_O_3_, with trivalent Bk, is in line with the relatively low redox potential of Bk(IV)/Bk(III) (+1.6 V in 1 M HClO_4_ versus NHE) compared to Cf(IV)/Cf(III) (+3.2 V versus NHE)^34,35^.

### Electronic structures of Bk and Cf

**Fig. 3:**
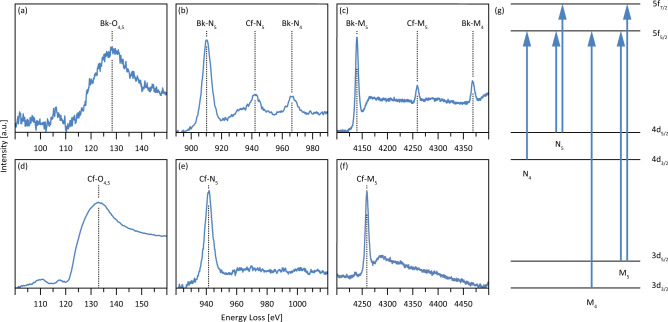
Electronic structures of Bk and Cf compounds. EELS spectra showing the **a** O_4,5_, **b** N_4,5_, and **c** M_4,5_ edges of Bk and the **d** O_4,5_, **e** N_4,5_, and **f** M_4,5_ edges of Cf. The spectra of Bk have contributions from Cf that formed by radioactive decay. Please note that the scaling of the energy loss axis differs depending on whether O_4,5_, N_4,5_, or M_4,5_ edges are shown. It is, however, consistent when comparing specific edges of the two elements. **g** Simple band diagram showing the transitions corresponding to the different white lines.

Absorption edges of the actinides have mostly been observed using synchrotron-based X-ray absorption spectroscopy (XAS), but only a handful of beamlines can accommodate radioactive materials^36^. Beside the logistical and administrative hurdles, even the microgram amounts required for XAS experiments often represent significant health hazards. EELS garners comparable data^24^, but excels at much lower energy ranges and requires significantly smaller sample amounts due to the increased beam-sample interactions of electrons compared to X-rays. Moore et al. successfully used EELS to study the O_4,5_, N_4,5_, and M_4,5_ edges of several bulk actinide metals up to Cm^1,25,27^ and we now contribute the O_4,5_, N_4,5_, and M_4,5_ edges of Bk and Cf (Fig. 3). Previously, only L edges of these two elements were measured using XAS. Initial measurements required hundreds of micrograms per sample^37–39^, although recent optimization reduced the required amount to 1–20 μg per XAS sample^40^. This is, however, still about three orders of magnitude higher than in our experiments.

The O_4,5_ edges measure 5d → 5f transitions, but both the O_4_ and the O_5_ edges are contained in a broad edge commonly referred to as the giant resonance^22^. The onsets for Bk (Fig. 3a) and Cf (Fig. 3d) are at energy losses of 110 and 121 eV, respectively. As the ^249^BkO_2_ sample also contained ~15% ^249^Cf due to the former’s radioactive decay, the measured O_4,5_ edge of Bk has a shoulder we ascribe to Cf. However, as the O_4,5_ edge of Bk is at a lower energy loss than that of Cf, its onset should be accurately determined. Both onsets also continue a trend established for the lighter actinides, where the onset shifts by approximately 10 eV from one actinide to the next^27^. Several peaks with a comparatively weak intensity precede the giant resonance peaks. These could have several causes, as described in the Supplementary Information, and we are therefore hesitant to assign physical meaning to them.

In contrast to the O_4,5_ edges, the N_4,5_ and M_4,5_ edges, which result from 4d → 5f and 3d → 5f transitions, respectively, have well-defined onsets and characteristic white lines (Fig. 3g). For Bk, the N_4,5_ (Fig. 3b) and M_4,5_ edges (Fig. 3c) have two well-defined white lines each. The N_5_ and the N_4_ edge have maxima at energy losses of 910 and 966 eV, respectively, and the M_5_ and the M_4_ edge have maxima at energy losses of 4140 and 4368 eV, respectively. In contrast, Cf only has one N_5_ white line (Fig. 3e) with a maximum at 942 eV and one M_5_ white line (Fig. 3f) with a maximum at 4259 eV but is missing N_4_ and M_4_ white lines. Due to the decay of ^249^Bk to ^249^Cf, the N_5_ and M_5_ white lines of Cf are also seen in the spectra of the Bk sample, but the edges are not expected to overlap with any features of the Bk edges. In spectra showing the M-edges (Fig. 3c, f), each of the white lines is followed by a second, rather broad peak at distances between 30 and 40 eV. These artifacts are likely due to multiple scattering processes involving plasmon excitations.

Of particular interest are the missing N_4_ and M_4_ edges of Cf, which normally result from the 4d_3/2_ → 5f_5/2_ and the 3d_3/2_ → 5f_5/2_ transitions, respectively (Fig. 3g). As electrons always transition from fully-filled d-bands, the intensities of these edges depend entirely on the occupancies of the f_5/2_ band and the absence of the N_4_ and the M_4_ edge consequently indicates a fully-filled f_5/2_ band. Similar behavior has been observed for metallic Cu^41,42^. Our results are in line with f-band occupancies calculated from the formal oxidation states that follow from the previously determined chemical compositions^43–47^: Cf(IV) with 5f^8^ in CfO_2_ and Cf(III) with 5f^9^ in Cf_2_O_3_. These findings also continue a trend observed by Moore et al., who described a steady decrease in the intensity of the N_4_ peak of the actinides until Am and attributed it to a filling of the 5f_5/2_ band under a *jj* coupling scheme (spin–orbit coupling > exchange interaction)^26^. Moore et al. also described a break in this behavior for Cm: Metallic Cm has an f-band occupancy of 5f^7^ and one would consequently expect a fully-filled 5f_5/2_ band and suppression of the N_4_ peak. Experiments contradicted this expectation, and they rationalized the unexpectedly intense N_4_ peak by a shift towards the *LS* coupling scheme (spin–orbit coupling < exchange interaction), leading to electrons filling the 5f_5/2_ as well as the 5f_7/2_ band. We have confirmed their results with oxidized Am and Cm compounds prepared using our experimental approach (Supplementary Figs. 6 and 7), and extend their line of reasoning to our findings. Bk, which in our samples has an expected configuration of 5f^7^ (Bk(IV) in BkO_2_) and therefore could fill the f_5/2_ band under a *jj* coupling scheme, behaves similarly to Cm and is shifted towards an *LS* coupling scheme, whereas Cf behaves like the early actinides and adheres to a *jj* coupling scheme.

Experimental observations made in the 1960’s and 1970’s^44–47^, and recently confirmed^10,11,48–50^, indicate that the transplutonium element series, unlike the analogous lanthanide series, is not monotonic. In particular, there is a growing body of evidence showing a pivot starting at Bk and Cf. Previous observations were made via solution thermodynamics experiments (complex formation constants and liquid–liquid extraction behavior), magnetic measurements, density functional theory (DFT) calculations, and structure determinations (extended X-ray absorption fine structure (EXAFS) and single crystal diffraction) with notably shorter-than-expected bond distances in the case of Cf compounds^5,10,11,40,50^.

### Conclusion and outlook

In this work, we demonstrated the comprehensive analysis of actinide-containing, crystalline particles using amounts, and thereby radioactivity levels, several orders of magnitude lower than with commonly employed methods. By working with such small amounts, we successfully reduced administrative and engineering controls, accelerated workflows, limited the radiological exposure to workers and equipment, used non-dedicated equipment, reduced self-irradiation artifacts, decreased the costs associated with materials procurement and waste disposal, and managed to work with heavily contaminated, yet precious, samples. The comparative study of the f-band occupancies of Bk and Cf yielded an unexpected change in the strength of the spin– orbit coupling relative to the exchange interaction and thereby further bolsters the status of Cf as a transitional element within the actinide series. Our approach can be extended to other radioactive materials and allows not only complementing prior studies of the early actinides, but also opens an avenue for exploring the solid-state chemistry of elements such as einsteinium (^252^Es and ^254^Es, respective half-lives: 472 and 276 days), fermium (^257^Fm, half-life: 100 days), and mendelevium (^258^Md, half-life: 51 days) for the first time.

## Methods

### Hazardous materials precautions

The four actinide isotopes investigated in this study were ^243^Am (*t*_1/2_ = 7388 years, specific activity = 14.8 GBq/g), ^248^Cm (*t*_1/2_ = 34,000 years, specific activity = 0.16 GBq/g), ^249^Bk (*t*_1/2_ = 330 days, specific activity = 61 TBq/g), and ^249^Cf (*t*_1/2_ = 352 years, specific activity = 0.15 TBq/g). Further noteworthy is that ^243^Am reaches a secular equilibrium with ^239^Np (*t*_1/2_ = 2.356 days) within ~24 days and that ^249^Bk decays to ^249^Cf. All of these isotopes are highly radioactive, present serious health risks, and should be handled with caution.

Consequently, preparation of the stock solutions and dropcasting onto TEM grids took place in laboratories specifically dedicated to the safe handling of radioactive materials. Regular radiation protection standards were then followed for the transfer to the National Center for Electron Microscopy (NCEM).

As TEM samples were prepared with only 1 – 10 ng of each isotope, the activities of the finished samples were rather low. For reference, 1 ng of freshly purified ^243^Am has an activity of about 30 Bq, 1 ng of ^248^Cm one of 1.2 Bq, 1 ng of ^249^Cf one of 151 Bq, and 1 ng of ^249^Bk one of 59 kBq. The TEM samples are considered non-dispersible, and repeated tests indicated that even dropping a grid from a height of a few centimeters did not cause material to come loose. As the samples’ radioactivity levels are several orders of magnitude below the applicable thresholds given in the Department of Energy (DOE) guideline 10 CFR 835, Appendix E, microscope rooms are not designated as “radioactive material areas” and the samples can be analyzed on non-dedicated electron microscopes. While these particular thresholds only apply to DOE labs, we assume that similar guidelines are in place at other institutions.

We nonetheless took a conservative approach and implemented several safety precautions at NCEM. All doors and work areas were labeled to indicate that radioactive materials were in use and that radiochemistry training was required to access them. The tables where the samples were mounted were covered with paper sheets to prevent contamination should a sample drop. Geiger counters (alpha and beta probes) were used to survey all work areas and all tools prior to and after working with samples. Each isotope had a dedicated set of tweezers to prevent cross-contamination. Further, we isolated the samples from the holders by using clips and washers dedicated to working with radioactive materials.

### Sample preparation

Chemically purified Am, Cm, Bk, and Cf salts from evaporated HCl solutions were purchased from Oak Ridge National Laboratory (ORNL). The ^248^Cm starting material had an isotopic purity of 95.78% (4.12% ^246^Cm, 0.06% ^245^Cm, 0.02% ^244^Cm, and 0.02% ^247^Cm isotopic distribution by atom percentage). The ^249^Bk starting material contained <0.05 ppm of ^252^Cf. Due to the radioactive decay of ^249^Bk into ^249^Cf, the ^249^Bk/^249^Cf-ratio at the time of the electron microscopy measurements (i.e., 65 days after the Bk/Cf separation performed at ORNL) was 6.84.

To prepare stock solutions for dropcasting, actinide chloride salts were dissolved in 0.1 M HCl (Standard, VWR Chemical BDH). The stock solutions were diluted with Milli-Q water to the μM level. TEM grids with ultrathin carbon films (Ted Pella Inc.) were held with dedicated negative-action tweezers and drops between 0.2 and 0.8 μL in volume were deposited onto them and left to dry in air at room temperature. The evaporation of the drop took about 30 min and was observed using an optical microscope (Supplementary Fig. 1). Once dried, samples were packaged and safely transferred to the National Center for Electron Microscopy.

### Scanning electron microscopy

A FEI Helios G4 UX was used to investigate the samples after receiving them at the microscope facility and to identify compounds of interest. All grids were mounted into a STEM sample holder reserved for radioactive materials. To acquire secondary electron images using the in-lens detector, the microscope was run in the immersion mode and at acceleration voltages between 1 and 5 kV. Further, STEM images were acquired using the HAADF-STEM detector at an acceleration voltage of 20 kV. The software MAPS was used to prepare large-scale, high-resolution images by sequentially acquiring thousands of images and stitching them. This large-scale image acquisition had the beneficial effect of immobilizing carbon compounds and minimizing carbon deposition during the TEM measurements. The SEM images were then analyzed manually to identify different components, whose chemistry was then probed by acquiring EDS maps using an acceleration voltage of 20 kV and an EDAX Elect Octane Super spectrometer.

### Transmission electron microscopy

Three transmission electron microscopes were used in this study, all operated at 300 kV. All experimental results were confirmed using several measurements.

Morphology and composition were mostly investigated using a FEI Titan or a FEI Titan Themis. The FEI Titan is equipped with a Gatan Ultrascan 1000 CMOS camera, a Gatan STEM detector, and a Bruker SuperX EDS detector. The image-corrected FEI Titan Themis is equipped with a Ceta2 CMOS camera, two STEM detectors, and a Bruker SuperX EDS detector.

The TEAM 0.5 microscope, a double-corrected, monochromated FEI Titan was used to acquire high-resolution STEM images and EELS spectra. As we were not looking at fine features in the EELS spectra, an energy resolution of 0.9 eV (no monochromation) was used. M-edges were acquired at extraction voltages of 4000 and 4050 eV to prevent any overlap of the FEG extraction voltage effect with the M-edges of Bk or Cf^1,2^. The TEAM 0.5 microscope is equipped with a special stage, a tilt-rotate design that allows full 360°-rotation range about both axes. This stage takes circular samples with a diameter of 1 mm, and parts of the grids were punched out and then glued into rings. In this way, the TEM holder is never directly in contact with the radioactive samples.

## Supplementary information

Supplementary Information

## Data Availability

All relevant images and spectra analyzed during this study are included in the article and its supplementary information. Raw data is available from the corresponding authors on request.
