# Supplementary material for: Probing electronic structure in berkelium and californium via an electron microscopy nanosampling approach
Source: Nat Commun. 2021 Feb 11;12:948. doi: 10.1038/s41467-021-21189-1 (PMC7878762; doi:10.1038/s41467-021-21189-1)
Supplement: Supplementary file 1 — Supplementary Information [file 41467_2021_21189_MOESM1_ESM.pdf]

Supplementary Information

Probing Electronic Structure in Berkelium and Californium via an Electron Microscopy Nanosampling Approach

Alexander Müller[1,2,#], Gauthier J.-P. Deblonde[3,4,#], Peter A. Ercius[1], Steven E. Zeltmann[1,2], Rebecca J. Abergel[3,5,*], Andrew M. Minor[1,2,*]

[1] *National Center for Electron Microscopy, Molecular Foundry, Lawrence Berkeley National Laboratory, Berkeley, CA 94720, USA*

[2] *Department of Materials Science and Engineering, University of California, Berkeley, CA 94720, USA*

[3] *Chemical Sciences Division, Lawrence Berkeley National Laboratory, Berkeley, CA 94720, USA*

[4] *Glenn T. Seaborg Institute, Physical & Life Sciences, Lawrence Livermore National Laboratory, Livermore, California 94550, USA*

[5] *Department of Nuclear Engineering, University of California, Berkeley, CA 94720, USA*

* *Corresponding authors. E-mails: aminor@lbl.gov (A.M.M.), rjabergel@lbl.gov (R.J.A.)*

[#] *These authors contributed equally: Alexander Müller, Gauthier J.-P. Deblonde*

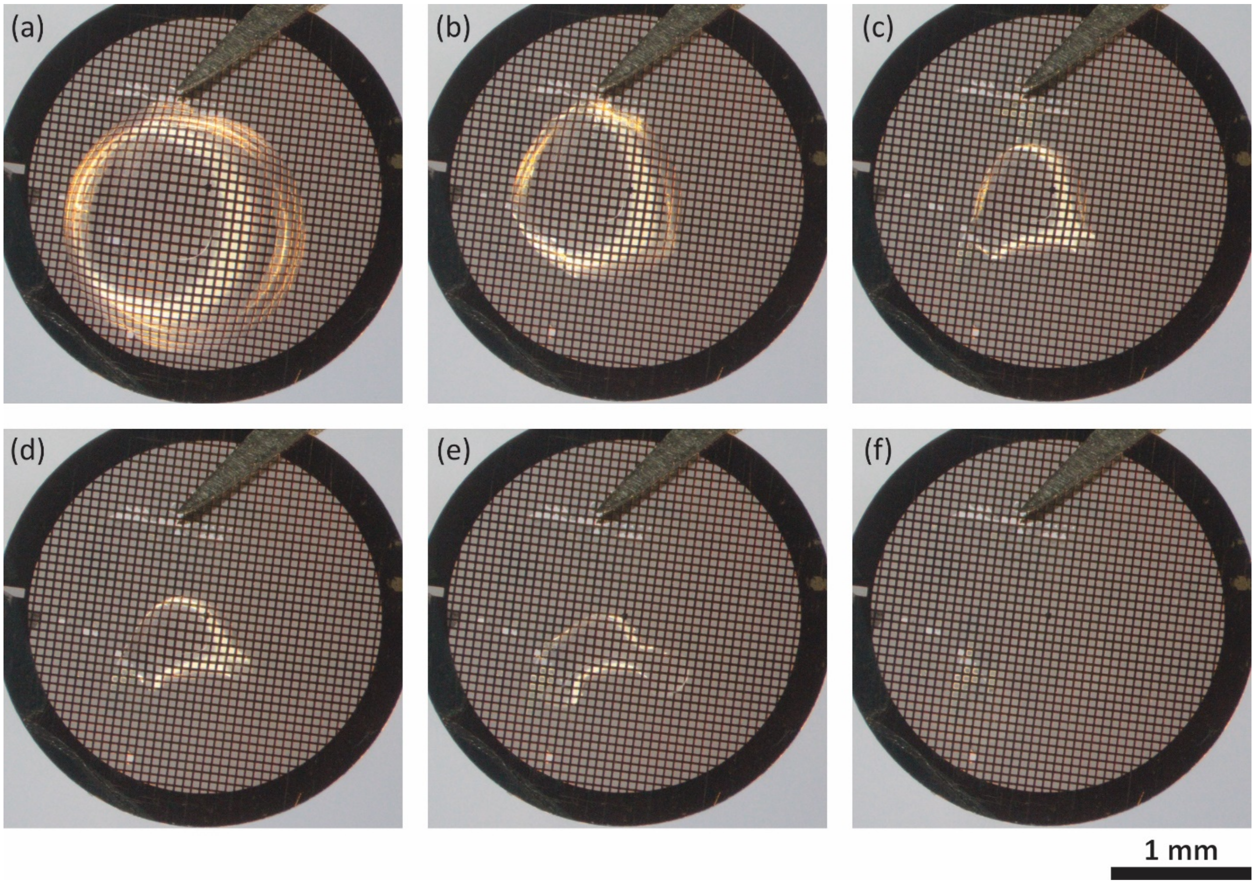

Supplementary Figure 1: **Photographs taken during the dropcasting process.** *These photographs were taken after depositing a 0.8 µL drop containing 1 ng Cm onto a TEM grid. (a) was taken right after dropcasting, (b)* after *8 minutes, (c) after 11 minutes, (d) after 12 minutes, (e) after 13 minutes, and (f) after 14 minutes.*

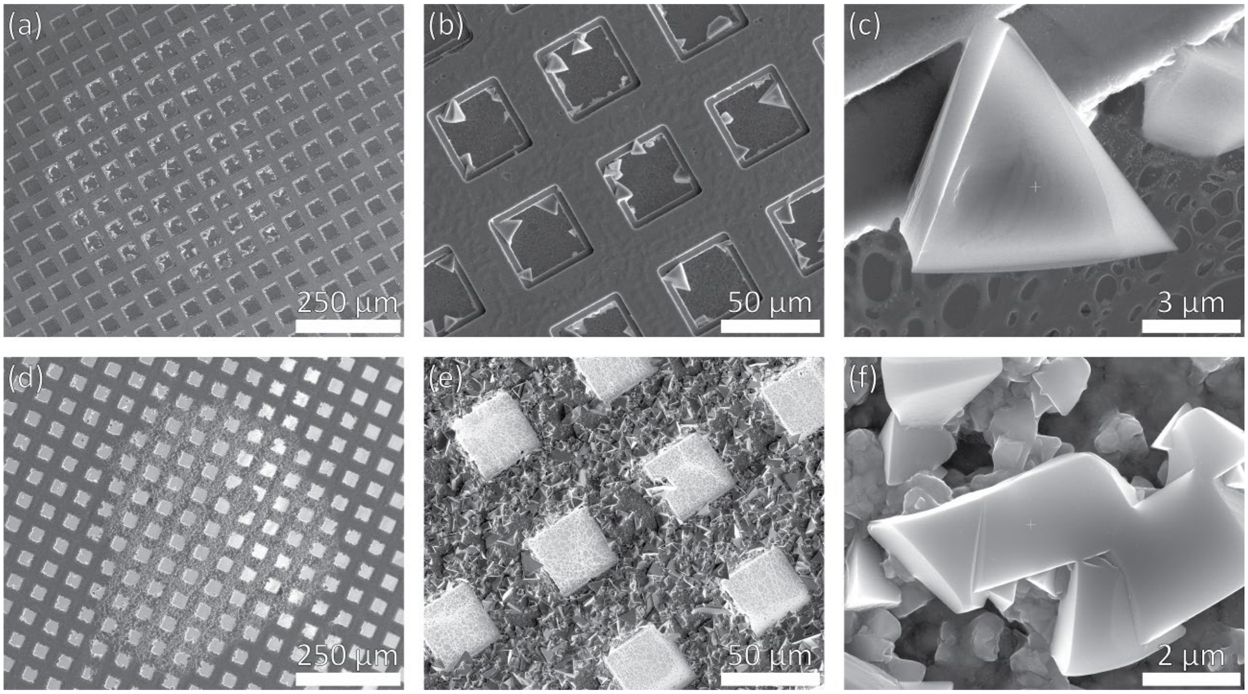

Supplementary Figure 2: ***Preliminary experiments with samarium chloride.*** *(a), (b) and (c) show scanning electron micrographs acquired after dropcasting a $SmCl_3$ solution onto the carbon side of a TEM grid. (d), (e) and (f) show micrographs acquired after dropcasting a $SmCl_3$ solution onto the gold side of a TEM grid.*

SUPPLEMENTARY DISCUSSION

Identification of Contaminants. One large source of contaminants found in the samples seemed to be the glassware used to prepare and transport the samples. After purification, the isotopes are in an acidic solution, which is collected in a glass vial and calcinated to eliminate the acid matrix as well as any volatile contaminants [3,4]. The residue can then be shipped more safely than a liquid, but we propose that this calcination introduces contaminants. However, until all sample handling steps are chemically investigated, this is only a hypothesis and the contaminations could also stem from any of the prior purification steps. The glass vials used to transport ^{249}Bk and ^{249}Cf to our laboratory contained the additives B_2O_3, Na_2O, K_2O, CaO, and MgO and we were able to identify some of these elements, as well as SiO_2 itself, using EDS and EELS (Supplementary Figure 3). Particularly common were cubic NaCl crystals and $CaCl_2$ needles.

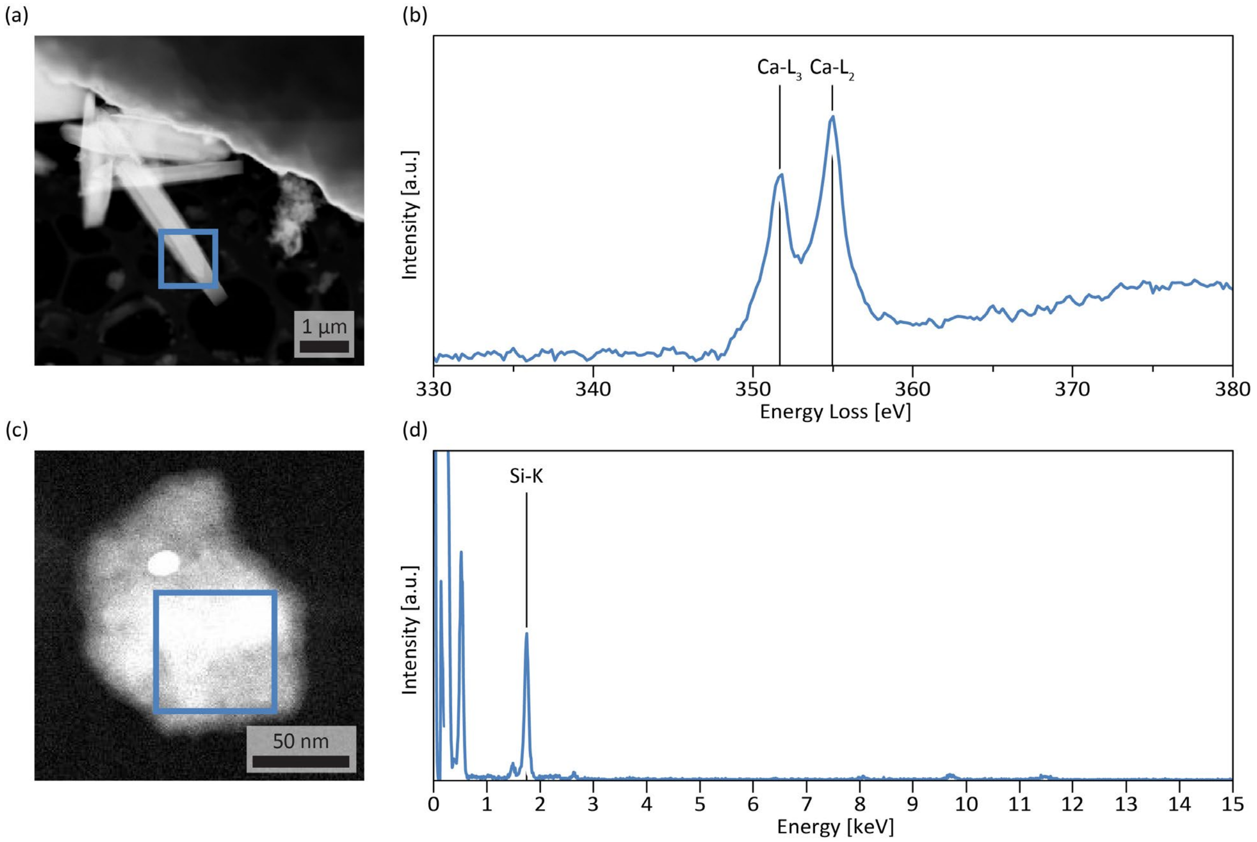

Supplementary Figure 3: ***Contaminants found in samples.*** *(a) Needles containing Ca as confirmed by EELS in (b). (c) SiO_2 particle as confirmed by EDS in (d). The areas of which the spectra were taken are marked with blue boxes.*

Another source of contaminants is linked to the purification and subsequent reuse of particularly rare isotopes such as ^{249}Bk and ^{249}Cf. In our case, amorphous carbon deposits, presumably from prior experiments involving organic ligands, dominated. We further found particles containing iron (Fe) and chromium (Cr), presumably from experiments involving steel labware, and gold (Au) nanoparticles (Supplementary Figure 4). Cerium (Ce), a fission product that forms during the production of ^{249}Bk and which is likely to follow Bk during the purification process owing to their similar ionic radii and redox properties [5], was also detected in low quantities relative to Bk (Supplementary Figure 4 f). Identifying these contaminants and precisely determining their locations allows selecting the desired compound amidst large amounts of contaminants, and the high spatial resolution of TEM is a large advantage. Further, by using annular dark-field mode in scanning TEM (STEM), in which the signal intensity is strongly linked to the average atomic number, the high atomic number of the actinides compared to the contaminants often makes differentiation between them trivial (Figure 1 c).

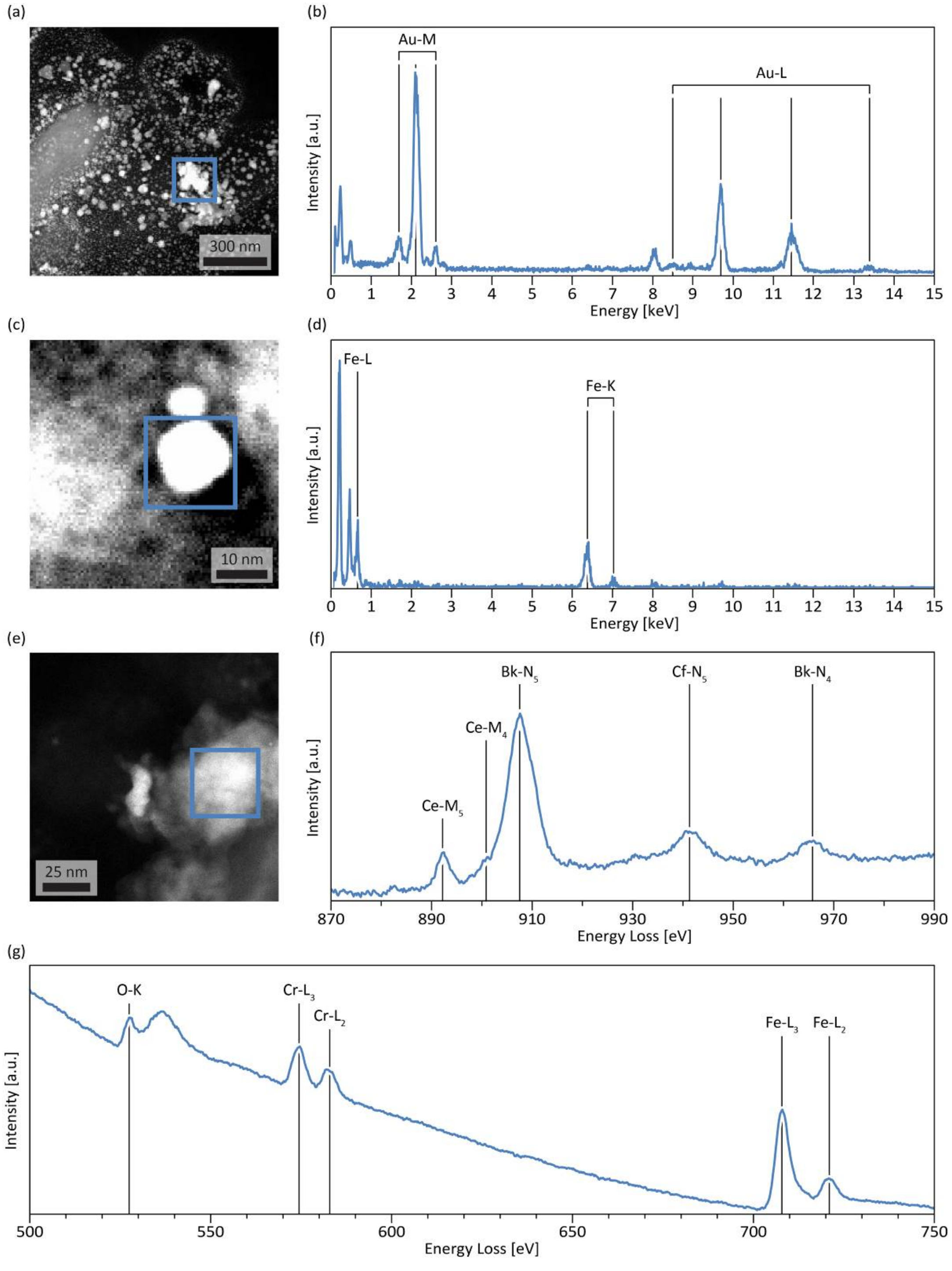

Supplementary Figure 4: ***Contaminants found in samples.*** *(a) Au nanoparticles as confirmed by EDS in (b). (c) Fe nanoparticle as confirmed by EDS in (d). (e) Region containing Bk and Ce as confirmed by EELS in (f). (g) EELS spectrum showing Cr and Fe – unfortunately, we did not acquire any images of the particle. The areas of which the spectra were taken are marked with blue boxes.*

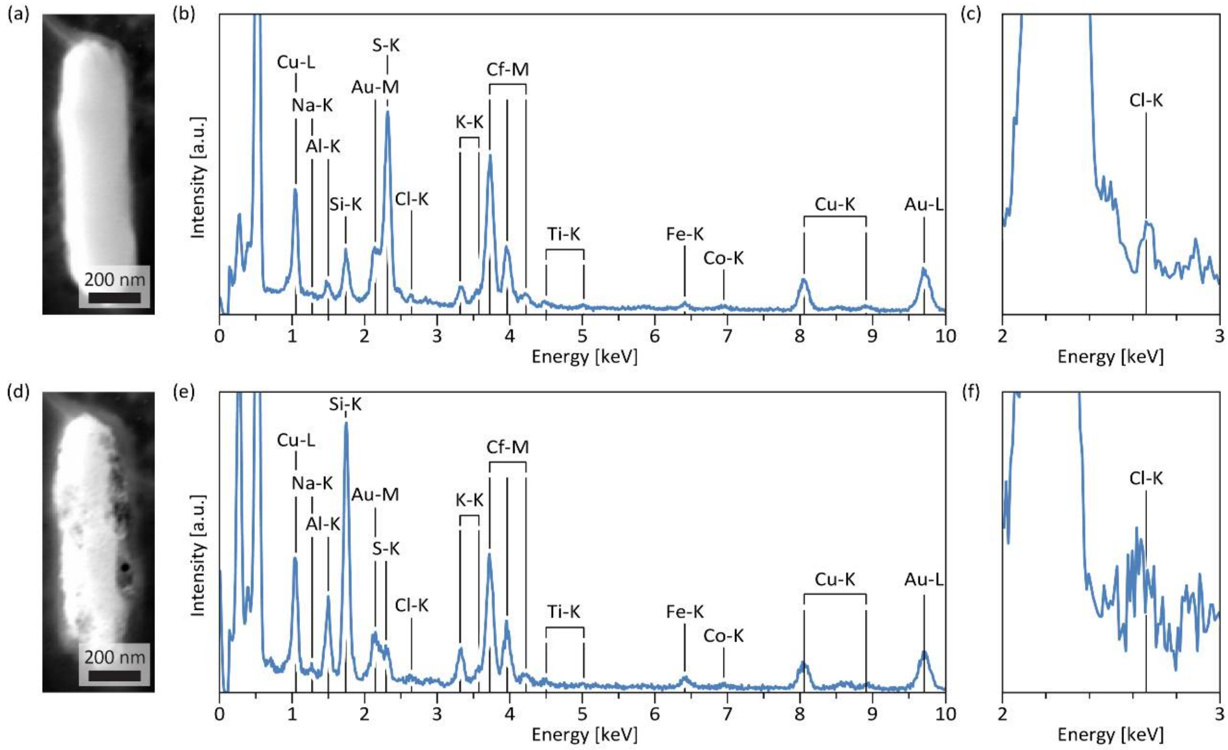

Supplementary Figure 5: ***Electron-beam-induced decomposition of a Cf-containing particle.*** *(a), (b) and (c) show STEM images and EDS spectra of a Cf-containing particle after minimal electron beam exposure. (d), (e) and (f) show STEM images and EDS spectra of the same particle after extensive electron beam exposure. Particularly of interest are Cl-K and S-K peaks, shown in (c) and (f), which are clearly visible before but not after electron beam exposure. The contaminant S was only found in this sample and we propose that it stems from the oxidizer sodium persulfate, which is used to separate Bk and Cf.*

Prepeaks of the $O_{4,5}$-Edges. Pre-peaks have been described to stem from ΔS = 1 transitions, which are normally dipole-forbidden but can be excited when spin-orbit coupling perturbs the electronic structure [6,7]. However, simulations of the $O_{4,5}$ edges by Butterfield *et al.* showed complex fine structures which are normally hidden by the giant resonance peak and which are not caused by ΔS = 1 transitions. As the position of the giant resonance peak is not well understood, it might not obscure such fine structure peaks. Lastly, we have shown the presence of contaminants such as cerium (Ce), which could also contribute to our measurements.

Am-/Cm-Containing Samples. In addition to the Bk- and Cf-containing samples discussed in the main text, we prepared similar samples containing Am and Cm.

In the Am sample, we observed large, Am-containing particles (Supplementary Figure 6 a) that were beam sensitive and quickly reacted under the electron beam to form Am_2O_3 (Supplementary Figure 6 b). EELS confirmed the presence of Am (Supplementary Figure 6 b). Both position and intensity ratio of the $N_{4,5}$ EELS edge were consistent with prior results [8–11].

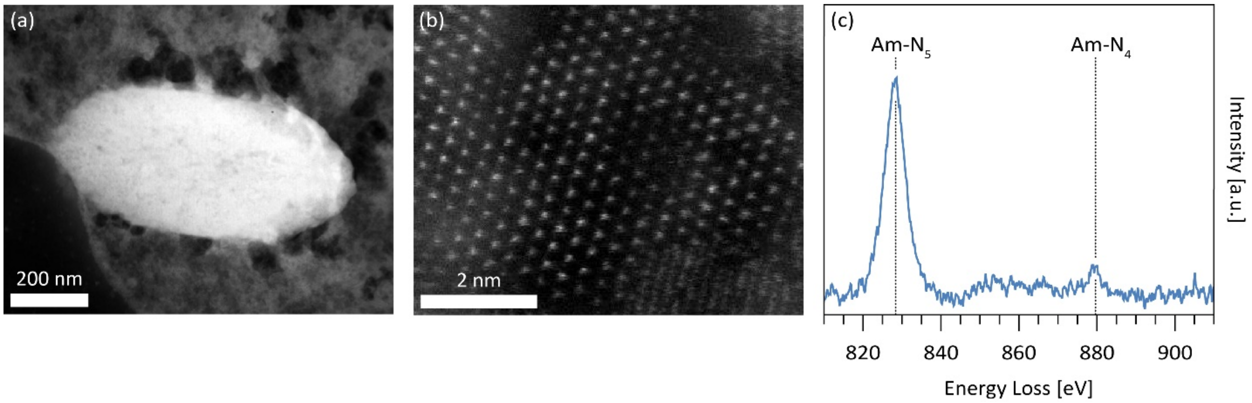

Supplementary Figure 6: ***TEM results acquired of an Am-containing sample.*** *(a) shows a large Am-containing particle in a SiO_2 matrix. The sample is changed by electron-beam irradiation and STEM images such as the one shown in (b) allow identifying the reaction product as Am_2O_3. (c) $N_{4,5}$ edge of Am_2O_3.*

Samples prepared using a $CmCl_3$ solution were significantly more contaminated than all others investigated in this study. Many regions were electron-opaque due to carbon buildup. In regions that were electron-transparent, we mostly observed needle-shaped $CaCl_2$ particles. These needles were extremely beam-sensitive (Supplementary Figure 7 a-d) and the electron beam drove the diffusion and agglomeration of individual atoms that had a very intense signal in STEM images (Supplementary Figure 7 e). EELS confirmed the presence of Cm in regions containing these atoms (Supplementary Figure 7 f), thereby indicating the formation of Cm-containing precipitates in a Ca-rich matrix. Unfortunately, even though these atoms formed crystalline lattices, we could not conclusively assign a known crystal structure to them.

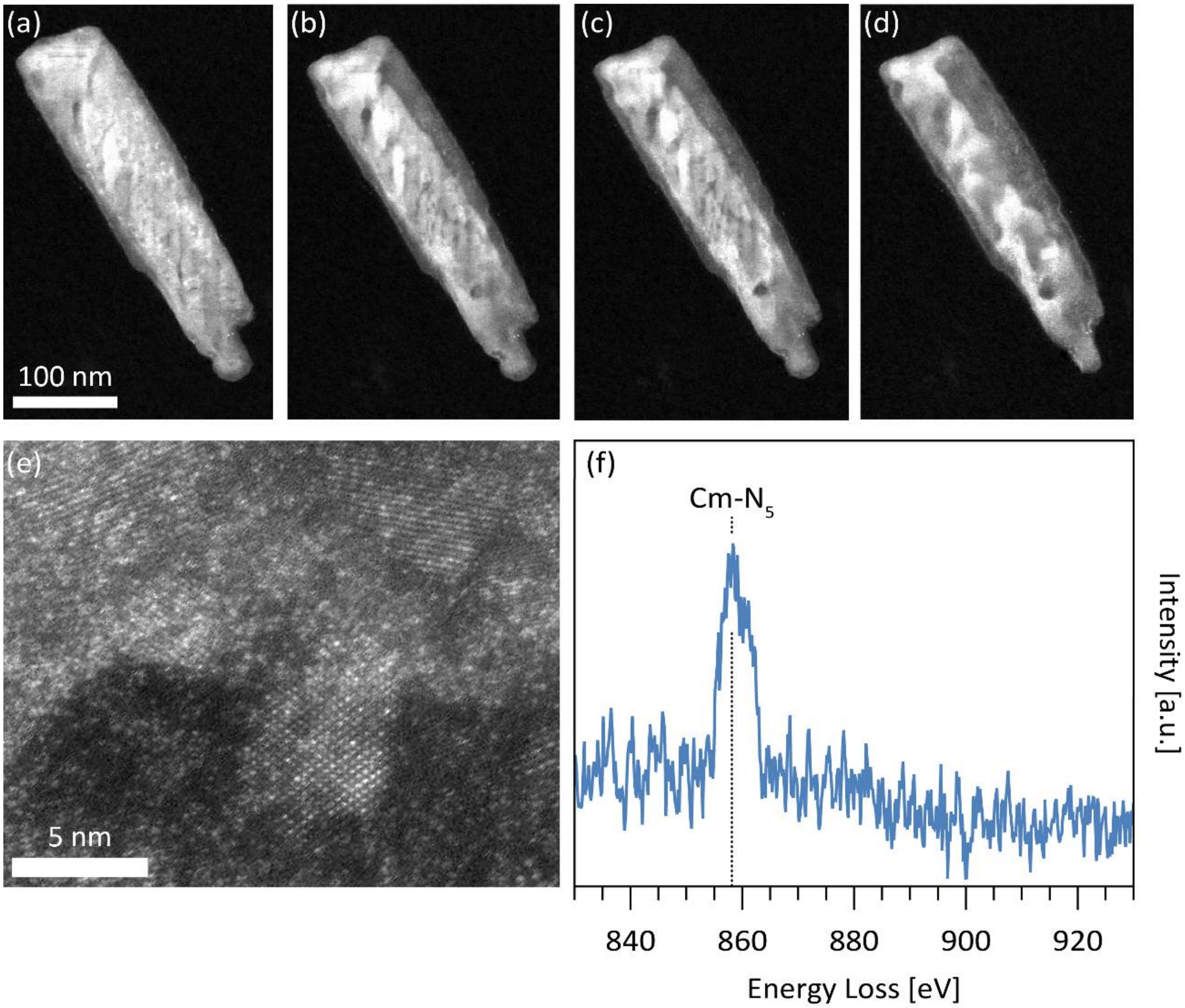

Supplementary Figure 7: ***TEM results acquired of an Cm-containing sample.*** *(a) – (d) show the electron beam irradiation-induced decomposition of a $CaCl_2$ needle. During this process, bright precipitates such as the one shown in (e) form. (f) These bright regions were identified as Cm-rich using EELS.*

Actinide EELS Edge Energies. To assess whether the edge energies we measured fit trends established by prior works, we compiled data on the $N_{4,5}$ and the $M_{4,5}$ edges from both literature and our own results (Supplementary Table 1). We chose to exclude $O_{4,5}$ edges from this comparison as their inherently complex fine structures make a consistent assessment of the onset, and thereby comparisons, difficult.

Within each row of the periodic table, the positions of EELS edges from one element to the next often follow trends. This holds true for most actinides (Supplementary Figure 8), with the sole

exception being the comparison between Cm and Bk. Excluding this step, the distances between N_5 and M_5 edges of one element to the next are (30.7 ± 2.1) eV and (113.4 ± 7.5) eV, respectively. In comparison, the distances between the N_5 and the M_5 edges of Cm and of Bk are 51 eV and 133 eV, respectively, and thereby roughly 20 eV larger than for the other actinides.

We also analyzed the distances between the $N_{4,5}$ and $M_{4,5}$ white lines of each element and trends in these distances from one element to the next (Supplementary Figure 8). The distance between the N_4 and the N_5 white lines increases by (2.6 ± 0.9) eV from one element to the next and the distance between the M_4 and the M_5 white lines rises by (9.9 ± 3.2) eV from one element to the next. This behavior was highly consistent and even for elements for which N_4 or M_4 white line have not yet been described (Cm and Cf), predicting their position should be straightforward.

Supplementary Table 1: ***Energy losses of the actinide N_4, N_5, M_4, and M_5 edges.*** *As the Cm-N_4, the Cf-N_4 and the Cf-M_4 edges have not yet been described, we could not yet include their positions here.*

**Z**	**Element**	**N_5 [eV]**	**N_4 [eV]**	**M_5 [eV]**	**M_4 [eV]**	**References**
90	Thorium	676	714	3332	3491	[12–14]
91	Protactinium	705	745	3453	3625	[15]
92	Uranium	738	780	3552	3728	[12,14,16]
93	Neptunium	770	816	3666	3850	[16–18]
94	Plutonium	801	850	3778	3973	[11,18,19]
95	Americium	828	879	3888	4092	This work, [8–11]
96	Curium	859	-	4007	4222	This work and [20]
97	Berkelium	910	966	4140	4368	This work
98	Californium	942	-	4259	-	This work

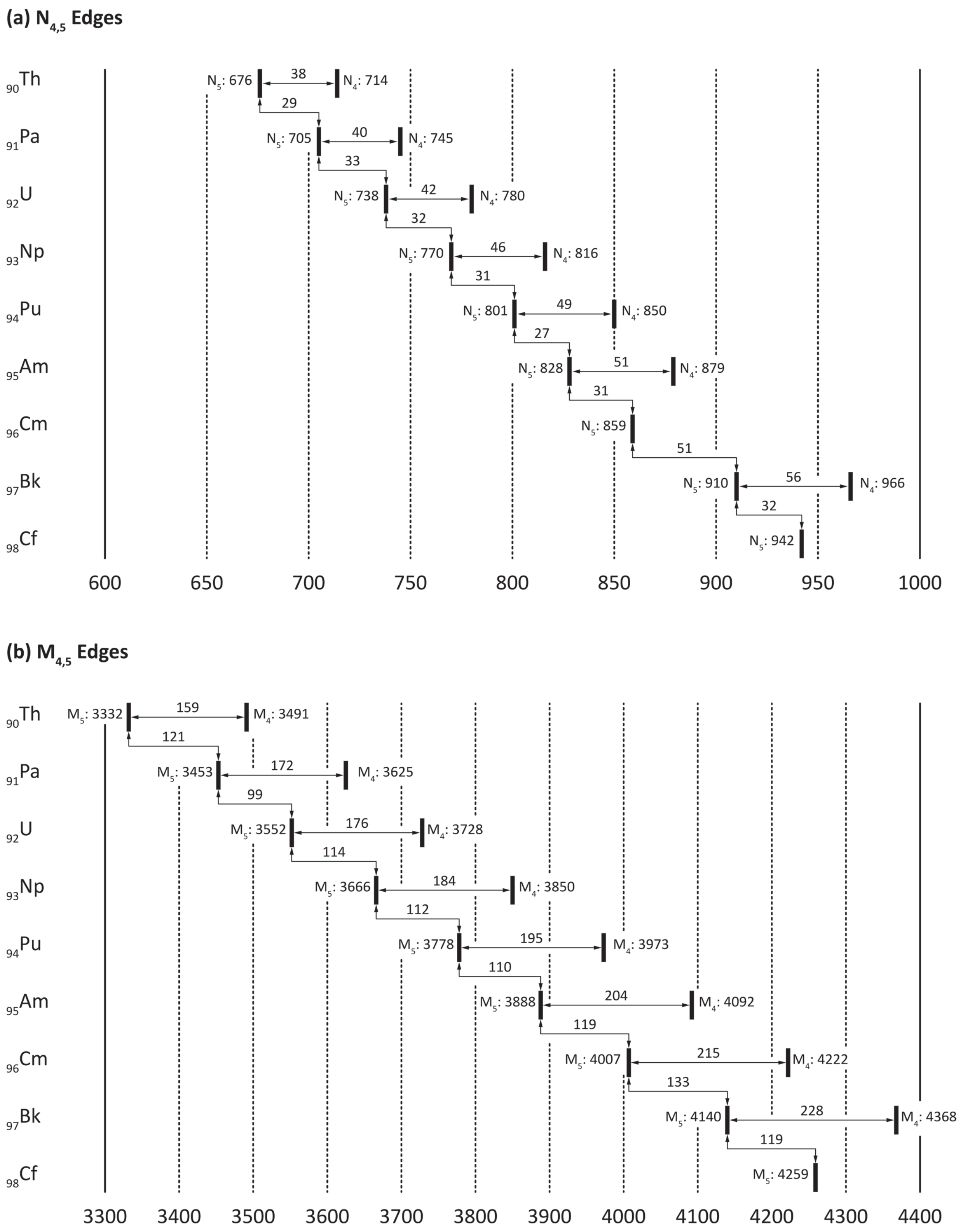

Supplementary Figure 8: ***Graphical plots of all known energy losses of the actinide $N_{4,5}$ and the $M_{4,5}$ edges.*** *(a) Graphical plot of all known energy losses of the actinide N_4 and N_5 edges. Distances*

between the N_4 and N_5 edges of each element are noted, as are the distances between the N_5 edges of one element to the next heavier one. (b) Graphical plot of all known energy losses of the actinide M_4 and M_5 edges. Distances between the M_4 and M_5 edges of each element are noted, as are the distances between the M_5 edges of one element to the next heavier one. As the Cm-N_4, the Cf-N_4 and the Cf-M_4 edges have not yet been described, we could not yet include their positions here.

Supplementary References

1. MacLaren, I., Annand, K. J., Black, C. & Craven, A. J. EELS at Very High Energy Losses. *Microscopy* **67**, i78–i85 (2018).

2. McComb, D. W. & Weatherly, G. C. The Effect of Secondary Electrons Generated in a Commercial FEG-TEM on Electron Energy-Loss Spectra. *Ultramicroscopy* **68**, 61–67 (1997).

3. Roberto, J. B. *et al.* Actinide Targets for the Synthesis of Super-Heavy Elements. *Nucl. Phys. A* **944**, 99–116 (2015).

4. Du, M., Tan, R. & Boll, R. Applications of *MP*-1 Anion Exchange Resin and Eichrom *LN* Resin in Berkelium-249 Purification. *J. Radioanal. Nucl. Chem.* **318**, 619–629 (2018).

5. Deblonde, G. J.-P. *et al.* Chelation and Stabilization of Berkelium in Oxidation State +IV. *Nat. Chem.* **9**, 843–849 (2017).

6. Moore, K. T. & Van Der Laan, G. Accurate Labeling of the Light-Actinide $O_{4,5}$ Edges. *Ultramicroscopy* **107**, 1201–1206 (2007).

7. Butterfield, M. T., Moore, K. T., Laan, G. Van Der, Wall, M. A. & Haire, R. G. Understanding the $O_{4,5}$ Edge Structure of Actinide Metals: Electron Energy-Loss Spectroscopy and Atomic Spectral Calculations of Th, U, Np, Pu, Am, and Cm. *Phys. Rev. B* **77**, 113109 (2008).

8. Wiss, T. *et al.* TEM Study of Alpha-Damaged Plutonium and Americium Dioxides. *J. Mater. Res.* **30**, 1544–1554 (2015).

9. Cross, J. N. *et al.* Covalency in Americium(III) Hexachloride. *J. Am. Chem. Soc.* **139**, 8667–8677 (2017).

10. Magnani, N. *et al.* Magnetic Polarization of the Americium J = 0 Ground State in $AmFe_2$. *Phys. Rev. Lett.* **114**, 097203 (2015).

11. Buck, E. C. & Fortner, J. A. Detecting Low Levels of Transuranics with Electron Energy Loss Spectroscopy. *Ultramicroscopy* **67**, 69–75 (1997).

12. Colella, M., Lumpkin, G. R., Zhang, Z., Buck, E. C. & Smith, K. L. Determination of the Uranium Valence State in the Brannerite Structure using EELS, XPS, and EDX. *Phys. Chem. Miner.* **32**, 52–64 (2005).

13. Jankovský, O. *et al.* Separation of Thorium Ions from Wolframite and Scandium Concentrates using Graphene Oxide. *Phys. Chem. Chem. Phys.* **17**, 25272–25277 (2015).

14. Ahn, C. C., Krivanek, O. L., Burgner, R. P., Disko, M. M. & Swann, P. R. *EELS-Atlas*. (Gatan, Inc., 1983).

15. Dieste, O. *et al.* TEM-EELS Analyses of Protactinium. *Mater. Res. Express* **6**, 26307 (2019).

16. Fortner, J. A., Finch, R. J., Kropf, A. J. & Cunnane, J. C. Re-Evaluating Neptunium in Uranyl Phases Derived from Corroded Spent Fuel. *Mater. Nucl. Syst.* **148**, 174–180 (2004).

17. Buck, E. C., McNamara, B. K., Douglas, M. & Hanson, B. D. *Possible Incorporation of Neptunium in Uranyl (VI) Alteration Phases*. (2003).

18. Buck, E. C. *et al.* Nature of Nano-Sized Plutonium Particles in Soils at the Hanford Site. *Radiochim. Acta* **102**, 1059–1068 (2014).

19. Fortner, J. A., Buck, E. C., Ellison, A. J. G. & Bates, J. K. EELS analysis of redox in glasses for plutonium immobilization. *Ultramicroscopy* **67**, 77–81 (1997).

20. Lander, G. H. *et al.* Measurements Related to the Magnetism of Curium Metal. *Phys. Rev. B* **99**, 224419 (2019).
